# Contribution of pathogenic T helper 1 and 17 cells to bursitis and tenosynovitis in polymyalgia rheumatica

**DOI:** 10.3389/fimmu.2022.943574

**Published:** 2022-08-11

**Authors:** Rosanne D. Reitsema, William F. Jiemy, Lieske Wekema, Annemieke M. H. Boots, Peter Heeringa, Minke G. Huitema, Wayel H. Abdulahad, Yannick van Sleen, Maria Sandovici, Caroline Roozendaal, Arjan Diepstra, Thomas Kwee, Bhaskar Dasgupta, Elisabeth Brouwer, Kornelis S. M. van der Geest

**Affiliations:** ^1^ Department of Rheumatology and Clinical Immunology, University of Groningen, University Medical Center Groningen, Groningen, Netherlands; ^2^ Department of Pathology and Medical Biology, University of Groningen, University Medical Center Groningen, Groningen, Netherlands; ^3^ Department of Laboratory Medicine, University of Groningen, University Medical Center Groningen, Groningen, Netherlands; ^4^ Medical Imaging Center, Department of Radiology, University of Groningen, University Medical Center Groningen, Groningen, Netherlands; ^5^ Department of Rheumatology, Southend University Hospital, Westcliff-on-Sea, United Kingdom

**Keywords:** polymyalgia rheumatica, T cells, T helper 1, T helper 17, ultrasound guided biopsy

## Abstract

**Background:**

Although polymyalgia rheumatica (PMR) is a very common rheumatic inflammatory disease, current insight into the pathobiology of PMR is limited and largely based on studies in blood. We investigated T helper 1 (T_H1_) and T helper 17 (T_H17_) cell responses in blood, synovial fluid and bursa tissue of patients with PMR.

**Materials and methods:**

Blood samples were collected from 18 patients with new-onset PMR and 32 healthy controls. Synovial fluid was aspirated from the inflamed shoulder bursae or biceps tendon sheath of 13 patients. Ultrasound-guided biopsies of the subacromial-subdeltoid (SASD) bursa were obtained from 11 patients. T cells were examined by flow cytometry, immunohistochemistry and immunofluorescence staining.

**Results:**

Besides an increase of T_H17_ (CD4^+^IL-17^+^IFN-γ^-^) cells and T cytotoxic 17 (T_C17_; CD8^+^IL-17^+^IFN-γ^-^) cells, no other major changes were noted in the circulating T cell compartment of patients with PMR. Absolute numbers of CD4^+^ and CD8^+^ T cells were similar in blood and synovial fluid of patients with PMR. Synovial fluid T cells showed an effector-memory (CD45RO^+^CCR7^-^) phenotype. Percentages of T_H1_ (CD4^+^IFN-γ^+^IL-17^-^) cells and T_H1_/T_H17_ (CD4^+^IFN-γ^+^IL-17^+^) cells, but not T_H17_ or T_C17_ cells, were increased in the synovial fluid. Bursa tissue biopsies contained a small number of T cells, which were mostly CD8 negative. The majority of bursa tissue T cells produced IFN-γ but not IL-17. For comparison, B cells were scarcely detected in the bursa tissue.

**Conclusion:**

Although the circulating T_H17_ cell pool is expanded in patients with PMR, our findings indicate that T_H1_ cells are involved in the inflammation of bursae and tendon sheaths in this condition. Our study points towards the T_H1_ cell pathway as a potential target for therapy in PMR.

## Introduction

Polymyalgia rheumatica (PMR) is the most common rheumatic inflammatory disease above the age of 50 (1). Patients typically present with pain and stiffness of the shoulder and pelvic girdle in association with an acute-phase response in the blood (1). Imaging studies have shown that inflammation of bursae and tendon sheaths is an essential feature of PMR (2–5), with subacromial-subdeltoid bursitis being the most common finding in ultrasound studies (6). PMR is also present in half of patients with giant cell arteritis, and PMR symptoms are a major cause of relapse in those patients (7, 8). Glucocorticoid treatment has remained the mainstay of treatment in PMR. Half of patients require prolonged glucocorticoid treatment (9), which is associated with substantial toxicity (10). Alternative targeted therapies for PMR are currently lacking, since little is known about the pathobiology of this condition.

Current insight into the pathobiology of PMR is mostly based on studies in blood (11, 12). An expansion of myeloid cells has been observed in the circulation of patients with PMR (13, 14). Serum IL-6 levels and numbers of circulating IL-6 producing B-cells are elevated in PMR (15–17). Increased frequencies of IL-17 producing CD4^+^ T cells (T helper 17 cells) have been reported in blood of PMR patients (18). A marked response by IFN-γ producing CD4^+^ T cells (T helper 1 cells), T helper 17 cells and IL-17 producing CD8^+^ T cells (T cytotoxic 17 cells) has been noted in the blood and arterial lesions of patients with giant cell arteritis (18–20). Tissue studies in PMR are rare, but an earlier study using arthroscopy showed that T cells may infiltrate the glenohumeral synovium of patients with longstanding PMR (21). It remains unclear whether and how T cells contribute to inflammation of bursae and tendon sheaths in PMR.

We hypothesized that T cells with a T_H1_ and T_H17_ signature promote shoulder inflammation in PMR. We investigated T cells in bursa/tenosynovial fluid and bursa tissue obtained from inflamed shoulders of patients with PMR, and determined whether these cells have a T_H1_ or T_H17_ cell phenotype.

## Materials and methods

### Patients

Peripheral blood was obtained from 18 patients with new-onset PMR prior to initiation of glucocorticoid treatment and from 32 age- and sex-matched healthy controls (HCs) (**Supplementary Table 1**). Healthy controls underwent medical history taking, physical examination, blood testing (including erythrocyte sedimentation rate) and urinalysis, as part of a study on healthy aging (22–24). Bursa fluid, tenosynovial fluid and/or bursa tissue were obtained from an additional cohort of 20 patients with active PMR, including new-onset and relapsing disease (**Supplementary Table 2**). The clinical diagnosis of PMR was made by expert rheumatologists and based on symptoms, physical examination, laboratory findings and, if available, imaging tests. The clinical diagnosis of PMR was confirmed in all patients after 6 months follow-up. In patients not fulfilling the EULAR/ACR classification criteria for PMR (5), the diagnosis of PMR was always confirmed by FDG-PET/CT. FDG-PET/CT was considered positive if the Leuven Score was ≥15, which is the optimal cut-off point for this composite FDG-PET/CT score in our cohort (2). The study was performed in accordance with the declaration of Helsinki. All patients provided written informed consent. The study was approved by the Medical Ethical Committee of the University Medical Center Groningen (METc 2010/222)

### Ultrasonography

Standard ultrasound images of the shoulder bursae and biceps tendon were obtained prior to aspiration of bursa or tenosynovial fluid and/or bursa tissue biopsy using a eSaote MyLabTwice with a LA533 (3-13 MHz) or LA435 (6-18 MHz) transducer. Power Doppler was used at the lowest permissible pulse repetition frequency with maximum color gain without creating artifacts. Bursitis was defined as an enlargement (i.e. increase in diameter) of the bursa, with a well-defined, anechoic or hypoechoic area inside, with or without power Doppler signal (25). Tenosynovitis was defined as an abnormal anechoic and/or hypoechoic tendon sheath widening which can be related both to the presence of tenosynovial abnormal fluid and/or hypertrophy, and power Doppler signal was considered positive if seen in two perpendicular planes within the peri-tendinous synovial sheath, excluding normal feeding vessels (26).

### Collection of synovial fluid and bursa tissue

Synovial fluid with paired blood samples were collected from 13 patients. Synovial fluid was aspirated from the subacromial-subdeltoid (SASD) bursa (n=9), subcoracoid bursa (n=1) or biceps tendon sheath (n=3). Ultrasound-guided biopsies of the SASD bursa were collected from 11 patients with a 16G or 18G core biopsy needle with a throw length of 13-23 mm (Argon Medical Devices) under local anesthesia with 1% lidocaine. Biopsies were dispersed in 10% formalin and paraffin embedded. Synovitis scores according to Krenn et al. were determined on hematoxylin and eosin (H&E)-stained sections (27).

### Flow cytometry

Absolute numbers of CD3^+^, CD4^+^ and CD8^+^ T cells and CD19^+^ B cells were determined in EDTA blood and synovial fluid using the MultiTest TruCount test (BD Biosciences) according to the manufacturer’s instructions. EDTA blood and synovial fluid were stained for 30 minutes with monoclonal antibodies recognizing the following surface markers: CD3, CD4, CD8, CD45RO and CCR7 (**Supplementary Table S3**). For experiments involving synovial fluid, an additional live/dead staining was performed using a fixable viability dye (ThermoFisher Scientific). Cells were fixed and red blood cells were lysed with FACS lysing solution (BD Biosciences). Cytokine production was assessed in heparin blood and synovial fluid. Blood and synovial fluid were stimulated with phorbol 12-myristate 13-acetate (PMA, 50 ng/mL) and calcium ionophore (1.6 µg/mL) for 4 hours at 37°C in the presence of Brefeldin A. Ammonium chloride was used to lyse red blood cells. Cells were stained for extracellular markers with monoclonal antibodies against CD3 and CD8. Cells were fixated and permeabilized with a Fix and Perm cell permeabilization kit (ThermoFisher Scientific). Intracellular markers were stained with monoclonal antibodies against CD4, IFN-γ, IL-17 and IL-4 (**Supplementary Table S3**). Samples were measured on a LSR-II flow cytometer (BD Biosciences) and analysed with Kaluza software v2.1 (Beckman Coulter). Unstimulated controls and appropriate fluorescence minus one (FMO) controls were used to determine setting of gates.

### Immunohistochemistry

Immunohistochemistry (IHC) was performed to detect CD3, CD8, CD20, CD68, IFN-γ and IL-17 (**Supplementary Table S4**). Sections were deparaffinized with xylene and dehydrated with alcohol before antigen retrieval was performed (pH9). Endogenous peroxidase was blocked prior to primary antibody incubation of 1 hour. Slides were subsequently incubated with secondary antibodies. Next, slides were incubated with DAB or AEC for 10 minutes and counterstained with hematoxylin. IHC sections were scanned with a Nanozoomer Digital Pathology Scanner (Hamamatsu Photonics) and analysed with QuPath software (version 0.2.3). IHC staining was semi-quantitatively scored on a five point scale: 0= no positive cells, 1= occasional positive cells (0–1% estimated positive), 2= small numbers of positive cells (>1–20%), 3= moderate numbers of positive cells (>20–50%), 4= large numbers of positive cells (>50%). Scores of two independent investigators were averaged.

### Immunofluorescence

Double labeling of CD3/IFN-γ and CD3/IL-17 was performed by immunofluorescence staining on biopsies of three patients. After deparaffinization and antigen retrieval, the tissues were incubated overnight with primary antibodies (**Supplementary Table S5**). Tissues were subsequently incubated with secondary and tertiary antibodies. Nuclei were stained using DAPI. Leica DFC345 FX was used to take images of the biopsies. Image cubes were captured at 40x magnification with Nuance Multispectral Imaging system v3.0.1 (PerkinElmer).

### Statistics

Non-paired analysis was performed by the Mann Whitney U test and paired analysis by the Wilcoxon Signed Rank test. P values <0.05 were considered statistically significant. Data were analyzed with GraphPad Prism 9.2.0.

## Results

### Increase of T_H17_ and T_C17_ cells in blood of patients with PMR

**Figure 1:**
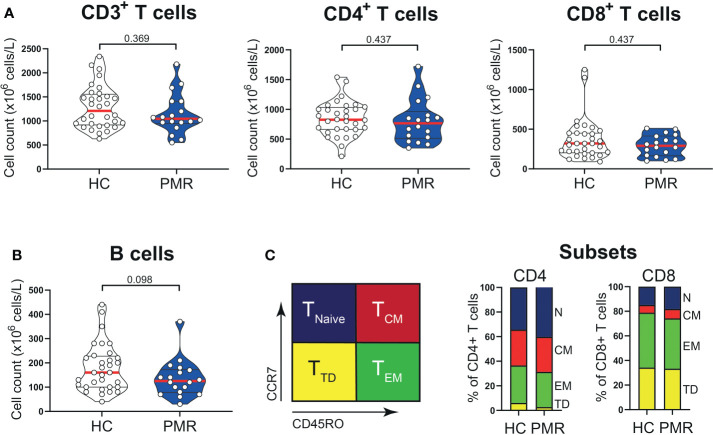
T cells in peripheral blood compartment of patients with PMR. **(A)** Absolute numbers of CD3^+^ T cells, CD4^+^ T cells, CD8^+^ T cells and **(B)** CD19^+^ B cells in peripheral blood of 18 patients with PMR and 32 healthy controls (HC). **(C)** Schematic overview (left panel) of flow cytometric gating for CD45RO^-^CCR7^+^ naive (T_Naive_), CD45RO^+^CCR7^+^ central memory (T_CM_), CD45RO^+^CCR7^-^ effector memory (T_EM_) and CD45RO^-^CCR7^-^ terminally differentiated (T_TD_) cells and percentages (right panel) of CD4^+^ and CD8^+^ T_Naive_, T_CM_, T_EM_ and T_TD_ cells in 18 patients with PMR and 32 healthy controls. Statistical significance by Mann-Whitney U test is indicated.

**Figure 2:**
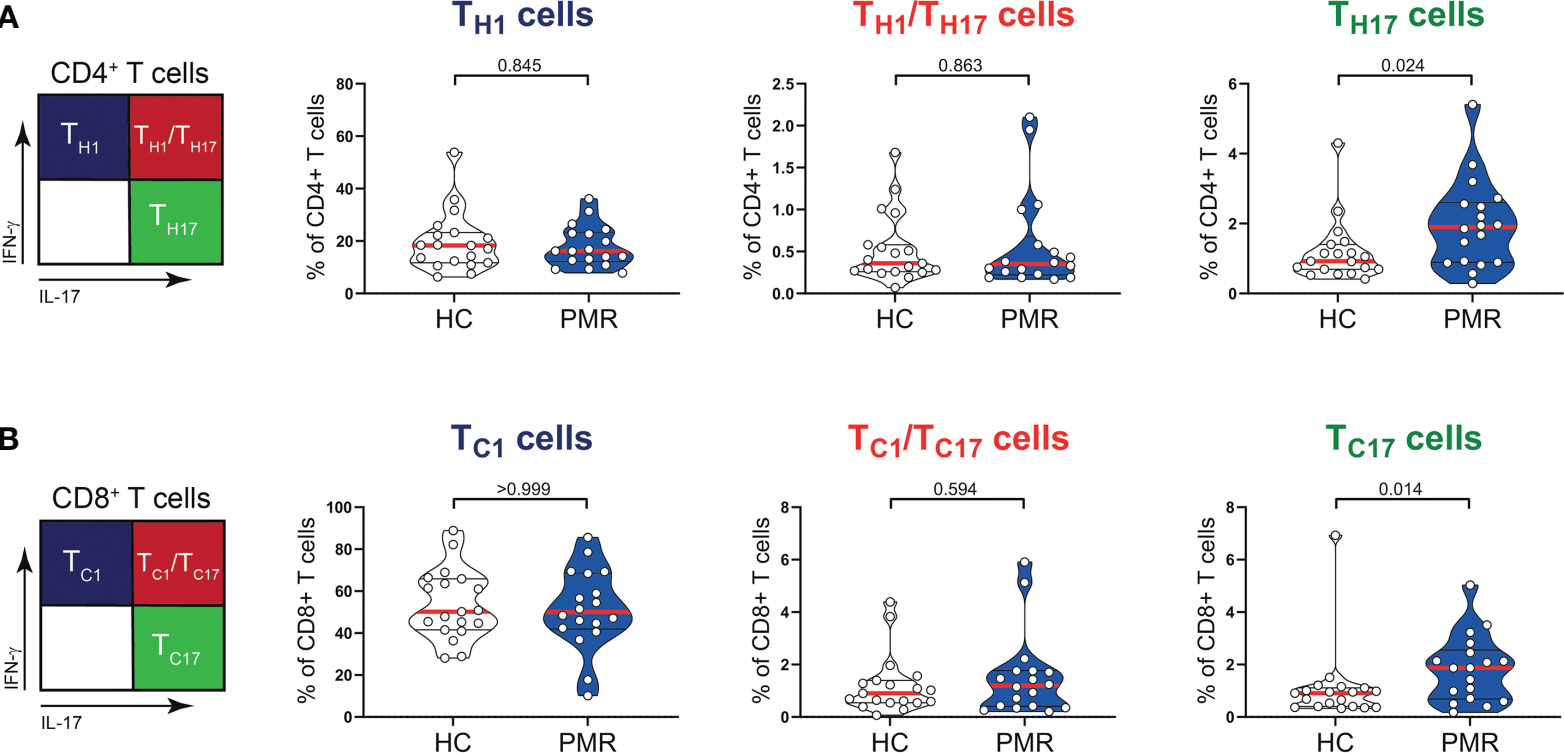
IFN-γ and IL-17 production by T cells in peripheral blood of patients with PMR. **(A)** Schematic overview (left panel) and percentages (right panel) of CD4^+^IFN-γ^+^IL-17^-^ (T_H1_), CD4^+^IFN-γ^+^IL-17^+^ (T_H1_/T_H17_) and CD4^+^IFN-γ^-^IL-17^+^ (T_H17_) T cells, and **(B)** CD8^+^IFN-γ^+^IL-17^-^ (T_C1_), CD8^+^IFN-γ^+^IL-17^+^ (T_C1_/T_C17_) and CD8^+^IFN-γ^-^IL-17^+^ (T_C17_) T cells in peripheral blood of 18 patients with PMR and 19 healthy controls (HC). Statistical significance by Mann-Whitney U test is indicated.

Numbers of circulating CD3^+^, CD4^+^, CD8^+^ T cells and also B cells were similar in patients with PMR and HCs (**Figures 1A, B**). Percentages of naive (T_Naive_), central memory (T_CM_) and effector memory (T_EM_) T cells were also comparable in patients and HCs (**Figure 1C**; **Supplementary Figure 1**), but the percentage of CD4^+^ terminally differentiated (T_TD_) T cells was slightly lower in patients than controls. No differences were observed for the percentages of CD4^+^ T helper 1 (T_H1_) cells, T_H1_/T_H17_ cells (**Figure 2A**) and IL-4 producing T_H2_ cells (**Supplementary Figure 2**). The same was true for their CD8^+^ counterparts (28): i.e. T cytotoxic 1 (T_C1_) cells, T_C1_/T_C17_ cells (**Figure 2B**) and T_C2_ cells (**Supplementary Figure 2**). However, the proportions of T_H17_ cells and T_C17_ cells were increased in the blood of patients with PMR. These findings indicate that the circulating T cell pool of patients with PMR is characterized by an enhanced IL-17 response.

### Enrichment of T_H1_ and T_H1_/T_H17_ cells in synovial fluid of patients with PMR

**Figure 3:**
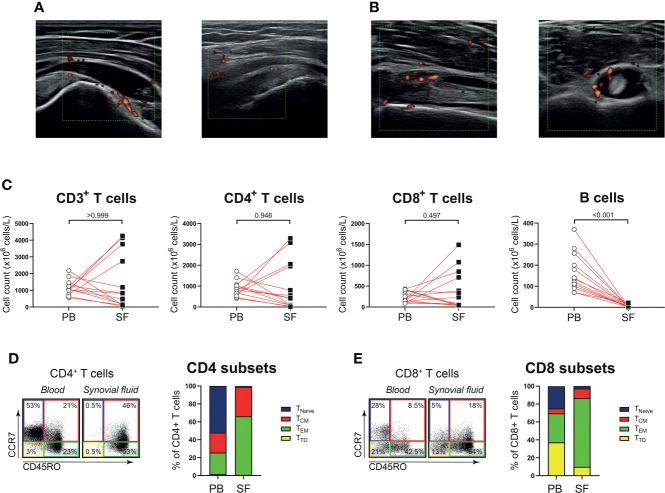
T cells in synovial fluid compartment of patients with PMR. **(A)** Representative ultrasound images of the inflamed subacromial-subdeltoid bursa of two patients with PMR. **(B)** Representative ultrasound images of the inflamed biceps tendon sheath of a patient with PMR, with the biceps tendon in longitudinal view (left panel) and transverse view (right panel). **(C)** Absolute numbers of CD3^+^ T cells, CD4^+^ T cells, CD8^+^ T cells and CD19^+^ B cells in peripheral blood (PB) and synovial fluid (SF) of 13 patients with PMR. **(D)** Representative flow cytometric staining (left panel) and percentages (right panel) of CD45RO^-^CCR7^+^ naive (T_Naive_), CD45RO^+^CCR7^+^ central memory (T_CM_), CD45RO^+^CCR7^-^ effector memory (T_EM_) and CD45RO^-^CCR7^-^ terminally differentiated (T_TD_) CD4^+^ T cells and **(E)** CD8^+^ T cells in PB and SF of 9 patients with PMR. Statistical significance by Wilcoxon signed rank test is indicated.

**Figure 4:**
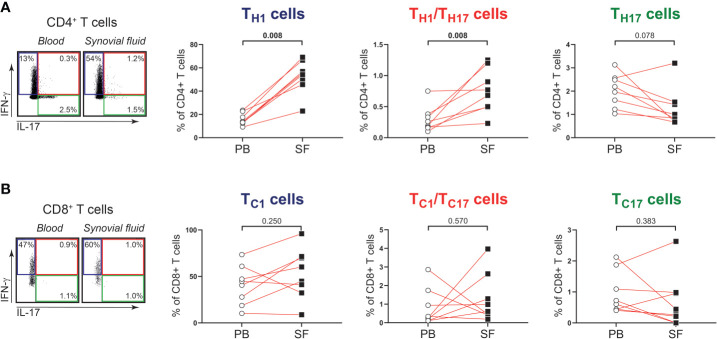
IFN-γ and IL-17 production by T cells in synovial fluid of patients with PMR. **(A)** Representative flow cytometric staining (left panel) and percentages (right panels) of CD4^+^IFN-γ^+^IL-17^-^ (T_H1_), CD4^+^IFN-γ^+^IL-17^+^ (T_H1_/T_H17_) and CD4^+^IFN-γ^-^IL-17^+^ (T_H17_) T cells, and **(B)** CD8^+^IFN-γ^+^IL-17^-^ (T_C1_), CD8^+^IFN-γ^+^IL-17^+^ (T_C1_/T_C17_) and CD8^+^IFN-γ^-^IL-17^+^ (T_C17_) T cells in peripheral blood (PB) and synovial fluid (SF) of 8 patients with PMR. Statistical significance by Wilcoxon signed rank test is indicated.

Next, we determined whether the phenotype and function of T cells in shoulder synovial fluid might be different from that in the blood of patients with PMR. Synovial fluid was obtained from shoulder bursae and tendon sheaths showing an effusion and synovial hypertrophy with power Doppler signal on ultrasound examination (**Figures 3A, B**). Synovial fluid T cell counts were similar to those in the blood, whereas B cells were hardly detected in the synovial fluid (**Figure 3C**). Disease stage (i.e. new-onset or relapsing disease) showed no clear relationship with synovial fluid T and B cell counts, (**Supplementary Figure 3**). Phenotypical analysis revealed a strong enrichment for T_EM_ cells in the synovial fluid (**Figures 3D, E**; **Supplementary Figure 4**). Proportions of T_H17_, T_C1_, T_C17_ and T_C1_/T_C17_ cells were similar in blood and synovial fluid (**Figures 4A, B**), as were percentages of T_H2_ and T_C2_ cells (**Supplementary Figure 5**). However, proportions of T_H1_ cells and T_H1_/T_H17_ were substantially higher in the synovial fluid than in the blood. Proportions of the various T_H_ and T_C_ cell subsets are shown in relation to PMR disease stage in **Supplementary Figure 6**. Taken together, T cells show a T_H1_ signature rather than T_H17_ signature in synovial fluid of patients with PMR.

### T cells infiltrate the synovial tissue of patients with PMR

**Figure 5:**
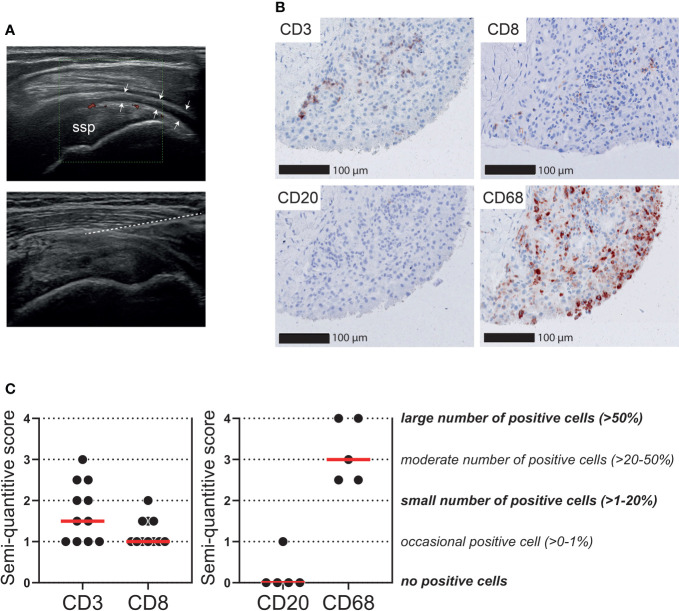
T cells in the bursa tissue of patients with PMR. Ultrasound-guided biopsies were obtained from the subacromial-subdeltoid (SASD) bursa of patients with PMR. **(A)** Representative ultrasound image (left panel) of inflamed SASD bursa (arrow heads), and trajectory of core biopsy needle (right panel) highlighted by the dashed line **(B)** Representative immunohistochemistry staining for CD3, CD8, CD20 (B cells) and CD68 (macrophages) in the synovial biopsy of a patient with PMR. **(C)** Semi-quantitative scoring for CD3 and CD8 in SASD bursa biopsies of 11 PMR patients (left panel), as well as CD20 and CD68 in 5 PMR patients (right panel). Scores of two independent investigators were averaged.

We further questioned to what extent T cell infiltrates are present in the bursa tissue of patients with PMR. Ultrasound-guided biopsies were obtained from the inflamed SASD bursa of 11 patients with active PMR (**Figure 5A**). Ultrasonographic evaluation of these bursae demonstrated synovial hypertrophy with the presence of power Doppler signal and varying amounts of synovial fluid. The number of biopsies obtained per patient was 5 (n=8), 3 (n=1) and 2 (n=2). As expected (29), all biopsies mainly consisted of connective tissue, while the synovial lining layer of the bursa was identified in 9/11 patients. In biopsies containing a synovial lining layer, the median synovitis score according to Krenn et al. was 2 (range: 0-5.5), which is suggestive of low-grade inflammation (27). Limited infiltrates of CD3^+^ T cells were detected in the biopsies (**Figures 5B, C**). Only an occasional CD8^+^ cell was found (0-1% of cells), which indicated that the majority of infiltrating T cells were in fact CD4^+^ T cells. For comparison, macrophages were the most abundant immune cell throughout the biopsies, whereas B cells were basically absent. Positive control staining is shown in **Supplementary Figure 7**. Exclusion of two patients without a synovial lining layer in their biopsies slightly increased the median semiquantitative CD3 score from 1.5 to 2, but did not change the CD8 score. CD3 and CD8 scoring are shown in relation to PMR disease stage in **Supplementary Figure 8A**.

### T cells produce IFN-γ rather than IL-17 in PMR synovial tissue

**Figure 6:**
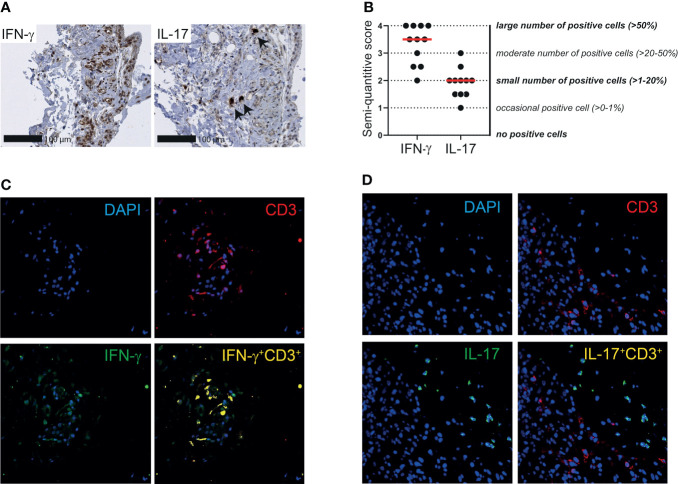
IFN-γ and IL-17 expression by T cells in synovial tissue of patients with PMR. **(A)** Representative immunohistochemistry staining for IFN-γ (left panel) and IL-17 (right panel) in the synovial biopsy of a patient with PMR. Arrows indicate IL-17^+^ cells. **(B)** Semi-quantitative scoring for IFN-γ and IL-17 in bursa tissue biopsies of 11 patients with PMR. The scores of two independent investigators were averaged. **(C)** Representative immunofluorescence staining for colocalization of IFN-γ and CD3 (yellow) in the bursa tissue biopsy of a patient with PMR. CD3 (red) and IFN-γ (green) single staining are also shown together with DAPI counterstaining (blue). **(D)** Representative immunofluorescence staining for colocalization of IL-17 and CD3 (yellow) in the bursa tissue biopsy of a patient with PMR. CD3 (red) and IL-17 (green) single staining are also shown together with DAPI counterstaining (blue). Representative immunofluorescence images of two other patients are shown in **Supplementary Figure S9**. Images are shown at 40x magnification.

Finally, we investigated the expression of IFN-γ and IL-17 in the synovial tissue of patients with PMR. A moderate to large number of IFN-γ producing cells was identified in the bursa tissue biopsies, whereas the number of IL-17 producing cells was small (**Figures 6A, B**). Similar results were obtained when the two patients without a synovial lining layer in their biopsies were excluded from the analysis. IFN-γ and IL-17 scoring in relation to PMR disease stage are shown in **Supplementary Figure 8B**. Co-staining of IFN-γ and CD3 in three patients suggested that most T cells in bursa tissue produce IFN-γ (**Figure 6C**; **Supplementary Figure 9A**). Other cellular sources of IFN-γ were also present, since not all IFN-γ producing cells were accounted for by CD3^+^ T cells. IL-17 producing cells were nearly uniformly CD3 negative (**Figure 6D**; **Supplementary Figure 9B**), which indicated that this cytokine is primarily produced by other cells in the bursa tissue. Taken together, our findings point towards involvement of the T_H1_ pathway in the inflammation of shoulder bursae and tendon sheaths in PMR.

## Discussion

**Figure 7:**
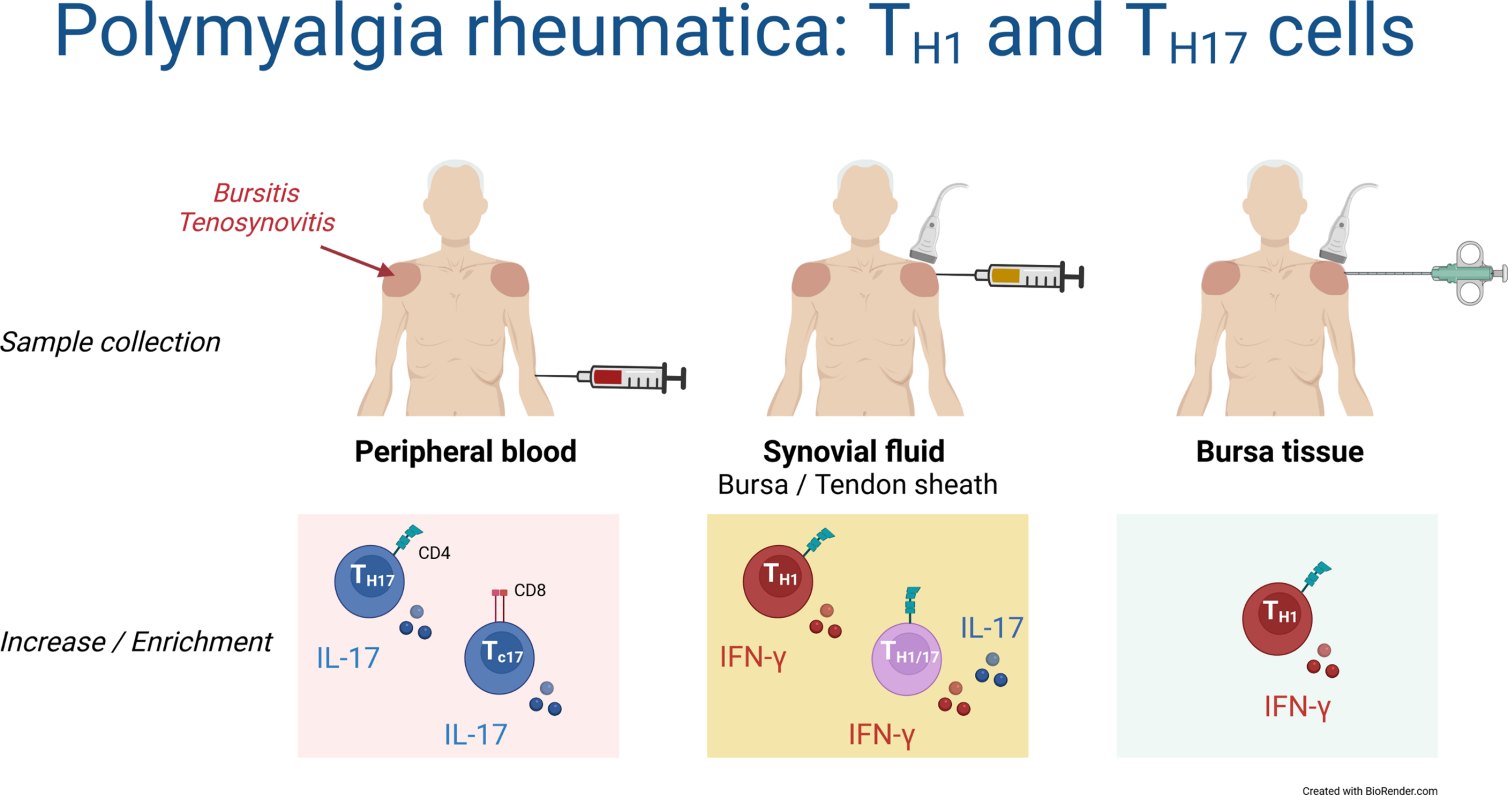
Schematic overview of T_H1_ and T_H17_ cells in blood, synovial fluid and bursa tissue of patients with PMR. Findings in the current study are shown.

This is the first study characterizing T cell responses in synovial fluid and bursa tissue of patients with PMR. Although findings in the blood suggested an important role of T_H17_ and T_C17_ cells in the pathobiology of PMR, we found no evidence for a prominent IL-17 response by T cells in the synovial fluid or bursa tissue. Instead, shoulder bursitis and tenosynovitis in PMR were characterized by a marked T_H1_ cell response (**Figure 7**).

The synovial fluid and tissue of patients with PMR were enriched with T_H1_ cells. Others previously observed a decrease of T_H1_ cells and an increase of T_H17_ cells in the blood of patients with PMR (18). We here confirm the expansion of the T_H17_ cells in the blood and report for the first time that their CD8^+^ T cell counterpart, i.e. T_C17_ cells, are also increased in the circulation of patients with PMR. Although T_H17_ and T_C17_ cells could play a role in the systemic inflammatory response of PMR, our study questions the direct contribution of these cells to the development of bursitis and tenosynovitis in patients with PMR. T_H1_ cells may potentially exert pro-inflammatory effects on macrophages, T cells and stromal cells in PMR lesions *via* their hallmark cytokine IFN-γ (30). T_H1_ cells have also been identified as key players in the vascular pathobiology of giant cell arteritis, where IFN-γ promotes the recruitment and activation of macrophages in arterial lesions (31). Although the pathogenic actions of IFN-γ in PMR await further investigation, the T_H1_ pathway could potentially be targeted *via* anti-cytokine therapies (e.g. anti-IFN-γ and anti-IL-12/23 therapy) or by blocking IFN-γ receptor signaling *via* inhibition of JAK1 and JAK2 (30).

Various mechanism might possibly explain the enrichment of T_H1_ cells in the synovial fluid and tissue. Firstly, it is likely that T cells directly migrate into the synovial tissue and are subsequently retained in the synovial fluid of patients with PMR. Most synovial fluid T cells were T_EM_ cells, which are equipped with chemokine receptors facilitating migration of these cells towards inflamed tissues (32). More specifically, circulating T_H1_ cells could migrate towards the synovial compartments under the influence of T_H1_ cell attracting chemokines, e.g. CXCL9 and CXCL10. Although the local expression of CXCL9 and CXCL10 remains to be investigated, enhanced production is suggested by elevated systemic levels of these chemokines in PMR (17, 33). Secondly, part of the synovial T_H1_ cells could be non-classic T_H1_ cells that develop from T_H17_ cells (34). Such plasticity has previously been observed in the synovial fluid of patients with juvenile idiopathic arthritis and rheumatoid arthritis (35, 36). Indeed, the small population of T_H1_/T_H17_ cells, which might be the transitional stage between T_H17_ and non-classic T_H1_ cells (35), was also increased in the synovial fluid. It would be interesting to further investigate and demonstrate such T cell plasticity in PMR *via* assessment of CD161 expression (37, 38).

Cells other than T cells seem to be responsible for the production of IL-17, and to some extent IFN-γ, in the synovial tissue of patients with PMR. Potential sources of IL-17 might include macrophages and mast cells, as indicated by synovial tissue studies in rheumatoid arthritis (39, 40). It might be possible that macrophages also secrete IFN-γ in PMR bursa tissue (41–43). Further studies are needed to elucidate the alternative cellular sources of IL-17 and IFN-γ in the bursa tissue of patients with PMR. In addition it would be interesting to study the expression of cytokines involved in T_H1_ and T_H17_ polarization, such as IL-12 and IL-1β/IL-6/IL-23, respectively. Moreover, complex cytokine networks in the bursa tissue may potentially include cytokines involved in macrophage polarization, such as GM-CSF and M-CSF. Indeed such cytokine networks have already been identified in the inflamed arteries of patients with giant cell arteritis (19, 44). Future studies on the cytokine environment in PMR tissue would ideally include both transcriptomic and proteomic analyses.

In contrast to T cells, B cells were essentially absent in the synovial fluid and bursa tissue of patients with PMR. This finding seems in agreement with an earlier study in which no B cells were found in the glenohumeral synovium of patients with PMR (21). Nevertheless, B cells might potentially contribute to the systemic inflammatory response in PMR. A prior study has shown that effector B cells are redistributed during active PMR, and that these cells promptly return to the circulation during remission while showing an enhanced capacity to produce IL-6 (16). A recent phase 2 study showed promising results of B cell depletion therapy in PMR (45, 46).

Based on imaging studies, it has been hypothesized that musculotendinous inflammation plays an important role in the pathobiology of PMR (47). Aging and mechanical stress have been postulated as potential contributors to such inflammation (48). In order to test these hypotheses, it would be interesting to extend the current tissue analyses to tendons and muscles of patients with PMR. As sampling of fragile tendons might prove difficult, novel imaging techniques with immune cell specific tracers could potentially aid the investigations of these structures in PMR (49).

A strength of our study is the unique investigation of T cells in three compartments (i.e. blood, synovial fluid and bursa tissue) of patients with PMR. We obtained synovial fluid and bursa tissue samples of key inflammatory lesions in PMR by minimal-invasive, ultrasound-guided approaches. The use of immunosuppressive treatments was very limited at the time of sampling. Our study also has limitations. Our sample size might have precluded the detection of less pronounced differences. It is recommended to obtain at least four ultrasound-guided biopsies for synovial studies (50), but this number of biopsies was not reached in four patients due to technical reasons and concern about the close proximity of the SASD bursa to the underlying supraspinate tendon. Nevertheless, it has been suggested that for assessment of T cell infiltrates less than four biopsies might potentially suffice (51). An investigation of the variability of the bursal tissue infiltrates could be of further interest. A synovial lining layer was not observed in biopsies of two patients, but exclusion of these patients did not substantially influence our findings in the bursa tissue. Control bursa tissue was not included in the current study. However, prior reports indicate that normal SASD bursa tissue only contains sporadic leukocytes located around small vessels, with most of these cells being macrophages rather than T cells (52, 53). T cells were only rarely detected in the SASD bursa tissue of patients with rotator cuff disease (54). Further comparison of bursa inflammation in PMR to that in other rheumatic inflammatory conditions could be of interest in future studies.

In conclusion, the bursitis and tenosynovitis of PMR are characterized by a marked T_H1_ response. Although T_H17_ cells are increased in the circulation, these cells are not enriched in the synovial fluid and bursa tissue of patients with PMR. Our study provides the first rationale for therapeutic targeting of the T_H1_ pathway in PMR.

## Data availability statement

All relevant data are contained within the article. The original contributions presented in the study are included in the article/**Supplementary Material**. Further inquiries can be directed to the corresponding author.

## Ethics statement

This study was reviewed and approved by METc UMCG. The patients/participants provided their written informed consent to participate in this study.

## Author contributions

BD, EB and KG contributed to conception of the work. RR, WJ, AB, PH, WA and KG contributed to design of the work. RR, WJ, LW, MH, YS, CR, AD, TK and KvdG contributed to acquisition of data. RR and KG contributed to analysis of data. RR, AB, PH, MS, EB and KvdG contributed to interpretation of data. All authors were involved in drafting of the work or revising it critically for important intellectual content. All authors provided final approval of the version published. All authors agreed to be accountable for all aspects of the work in ensuring that questions related to the accuracy or integrity of any part of the work are appropriately investigated and resolved.

## Funding

The study was supported by a research grant from FOREUM Foundation for Research in Rheumatology. The study was also funded by the Rheumatology Grant (Dutch Society for Rheumatology) and Mandema Stipend (University Medical Center Groningen).

## Acknowledgments

We thank the patients that participated in our study. We thank the staff of the Medical Immunology Department for their technical assistance. We thank Prof. R.H.J.A. Slart for evaluating the FDG-PET/CT scans.

## Conflict of interest

KG reports personal fees from Roche, outside the submitted work. BD reports consulting fees from Roche, Chugai, Sanofi, and sponsorship grants for international meetings and workshops with Roche, Sanofi, AbbVie and GlaxoSmithKline. EB reports personal fees from Roche, outside the submitted work.

The remaining authors declare that the research was conducted in the absence of any commercial or financial relationships that could be construed as a potential conflict of interest.

## Publisher’s note

All claims expressed in this article are solely those of the authors and do not necessarily represent those of their affiliated organizations, or those of the publisher, the editors and the reviewers. Any product that may be evaluated in this article, or claim that may be made by its manufacturer, is not guaranteed or endorsed by the publisher.
