# Supplementary material for: Contribution of pathogenic T helper 1 and 17 cells to bursitis and tenosynovitis in polymyalgia rheumatica
Source: Front Immunol. 2022 Aug 11;13:943574. doi: 10.3389/fimmu.2022.943574 (PMC9402989; doi:10.3389/fimmu.2022.943574)
Supplement: Supplementary file 1 [file DataSheet_1.pdf]

1. Supplementary Tables

Supplementary Table 1. Characteristics of subjects contributing to blood analyses of patients versus controls. Data are shown for 18 patients with new-onset PMR and 32 healthy controls that contributed to analyses presented in Figures 1 and 2. FDG-PET/CT = fluorodeoxyglucose positron emission tomography combined with low-dose computed tomography.

	**PMR patients (n=18)**	**Healthy controls (n=32)**
Sex, no. of females	12 (67%)	18 (56%)
Age, median (range)	69 (54-84)	71 (58-91)
Fulfilling EULAR/ACR criteria for PMR, no. of patients (%)	17 (94%) [a]	
FDG-PET/CT positive for PMR, no. of patients (%)	15 (83%) [b]	
Confirmation of PMR diagnosis after 6 months follow-up, no. of patients (%)	18 (100%)	
Exclusion of large vessel giant cell arteritis by imaging, no. of patients (%)	17 (94%) [c]	
C-reactive protein, mg/L, median (range)	42 (0.6-186) [d]	
Erythrocyte sedimentation rate, mm/hr, median (range)	48 (11-107)	10 (2-20)

[a] *One patient did not fulfil the EULAR/ACR classification criteria for PMR, but the diagnosis was confirmed by FDG-PET/CT and at least 1 year of follow-up. Classification criteria in this patient were not met due to a normal C-reactive protein level and erythrocyte sedimentation rate.*

[b] *FDG-PET/CT was performed in 15 patients. The FDG-PET/CT scan was considered positive if the Leuven Score was ≥15, i.e. the optimal cut-off point for this composite FDG-PET/CT score in our cohort (1).*

[c] *Imaging for large vessel giant cell arteritis was done by FDG-PET/CT and/or axillary artery ultrasonography. In one patient, no imaging for large vessel GCA was performed. This patient successfully tapered and stopped glucocorticoid treatment within 9 months after diagnosis, without any relapses or subsequent reactivation of disease.*

[d] *CRP was determined in 17 patients.*

Supplementary Table 2. Characteristics of patients with PMR providing bursa fluid, tenosynovial fluid and/or bursal tissue. Data are shown for 19 patients with active (new-onset and relapsing) PMR that contributed to analyses presented in Figures 3-6. Paired blood and bursa fluid / tenosynovial fluid were obtained from 13 patients: subacromial-subdeltoid bursa (n=9), biceps tendon sheath (n=3) and subcoracoid bursa (n=1). Bursa tissue samples were obtained from 11 patients. CRP = C-reactive protein. DMARD = disease modifying antirheumatic drug. ESR = erythrocyte sedimentation rate. FDG-PET/CT = fluorodeoxyglucose positron emission tomography combined with low-dose computed tomography.

**Patients' characteristics**		**Patients with PMR (n=19)**
**Sex**	*No. of females (%)*	14 (74%)
**Age**	*Median (range) years*	69 (58-83)
**Disease stage**	*New onset PMR, no. of patients (%)*	15 (79%)
	Relapsing PMR, no. of patients (%)	4 (21%)
**Fulfilling EULAR/ACR criteria for PMR**	*No. of patients (%)*	17 (90%) [a]
**FDG-PET/CT positive for PMR**	*Positive, no. of patients (%)*	8 (42%) [b]
	Negative, no. of patients (%)	2 (11%)
	Not done, no. of patients %)	9 (47%)
**Confirmation of PMR diagnosis after 6 months follow-up**	*No. of patients (%)*	19 (100%)
**Imaging for large vessel giant cell arteritis**	*Positive, no. of patients (%)*	0 (0%) [c]
	Negative, no. of patients (%)	19 (100%)
**Prednisolone treatment at time of sampling**	*No. of patients (%)*	1 (5%)
	Dose, mg	2.5
**DMARD use at time of sampling**	*Leflunomide or placebo, no. of patients (%)*	1 (5%) [d]
	Other DMARD, no. of patients (%)	0 (0%)
**Lab at time of sampling**	*ESR, mm/hour, median (range)*	54 (11-89)
	CRP, mg/L, median (range)	23 (6-118)

[a] Two patients did not fulfil the EULAR/ACR classification criteria for PMR, but their diagnosis was confirmed by FDG-PET/CT and at least 1 year of follow-up. Classification criteria were not met due to normal CRP and ESR in one patient, and the presence of mild synovitis of the left wrist and absence of morning stiffness> 45 min in the other patient. [b] FDG-PET/CT was performed in 10 patients. The FDG-PET/CT scan was considered positive if the Leuven Score was ≥15, i.e. the optimal cut-off point for this composite FDG-PET/CT score in our cohort (1). [c] Imaging for large vessel giant cell arteritis was done by FDG-PET/CT and/or axillary artery ultrasonography. [d] Patient was enrolled in a randomized-controlled trial and received either leflunomide or placebo.

Supplementary Table 3. Antibodies for flow cytometry.

Marker	Clone	Fluorochrome	Company
CD3	OKT-3	eFluor 605	eBioscience
CD3	SK7	BUV737	BD
CD4	RPA-T4	eFluor 450	eBioscience
CD4	RPA-T4	APC-H7	BD
CD8	SK-1	PerCP	BD
CD8	SK-1	PE-Cy7	Biolegend
CD45RO	UCHL-1	FITC	Biolegend
CCR7	3D12	PE-Cy7	BD
CCR7	3D12	AF747	BD
IFN-γ	4S.B3	PerCP-Cy5.5	Biolegend
IFN-γ	4S.B3	BV786	BD
IL-4	8D4-8	PE	Biolegend
IL-4	8D4-8	PerCP-Cy5.5	BD
IL-17a	eBio64DEC17	FITC	ThermoFisher

Supplementary Table 4. Antibodies for immunohistochemistry.

Marker	Antigen retrieval	Isotype	Clone	Dilution	Secondary antibody
CD3	pH9	Mouse IgG1	F7.2.38	1:50	Envision anti-mouse polymer-HRP (DAKO, K4006)
CD8	pH9	Rabbit monoclonal	EP1150Y	1:500	Envision anti-rabbit polymer (DAKO K4003) or Rabbit IgG VisUCyte HRP (R&D VC003)
CD20	pH9	Mouse IgG2a	L26	1:50	Envision anti-mouse polymer-HRP (DAKO, K4006)
CD68	pH9	Mouse IgG3	PG-M1	1:100	Envision anti-mouse polymer-HRP (DAKO, K4006)
IFN-γ	pH9	Mouse IgG2b	IFNG/466	1:500	Envision anti-mouse polymer-HRP (DAKO, K4006)
IL-17	pH9	Goat polyclonal	Polyclonal (Novus, AF-317-NA)	1:400	Goat IgG VisUCyte HRP (R&D VC004)

Supplementary Table 5. Antibodies for immunofluorescence.

Marker	Secondary antibody	Tertiary antibody	Conjugate/Dye
CD3 (Mouse IgG1, 1:20)	Rabbit anti-mouse IgG1 (Novus, NBP1-72793, 1:50)	Donkey anti-rabbit (ThermoFisher, a31572, 1:50)	AF555
IFN-γ (Mouse IgG2b, 1:250)	Rat anti-mouse IgG2b (Biolegend, RMG2b-1, 1:20)	Donkey anti-rat IgG (abcam, ab150153, 1:40)	AF488
IL-17 (Goat polyclonal, 1:400)	Donkey anti-goat (abcam, ab150129, 1:50)	-	AF488

2. Supplementary Figures

Supplementary Figure 1. T cell differentiation subsets in peripheral blood of patients with PMR and healthy controls. Percentages of naive (T_{Naive}), central memory (T_{CM}), effector memory (T_{EM}) and terminally differentiated (T_{TD}) $CD4^+$ T cells and $CD8^+$ T cells are shown for 18 patients with PMR and 32 healthy controls (HC). Median percentages for $CD4^+$ T_{TD} cells were 3.2% (range 0.6 to 49.1) in HC and 2.2% (range 0.4 to 8.8) in patients with PMR. Statistical significance by Mann-Whitney U test is indicated.

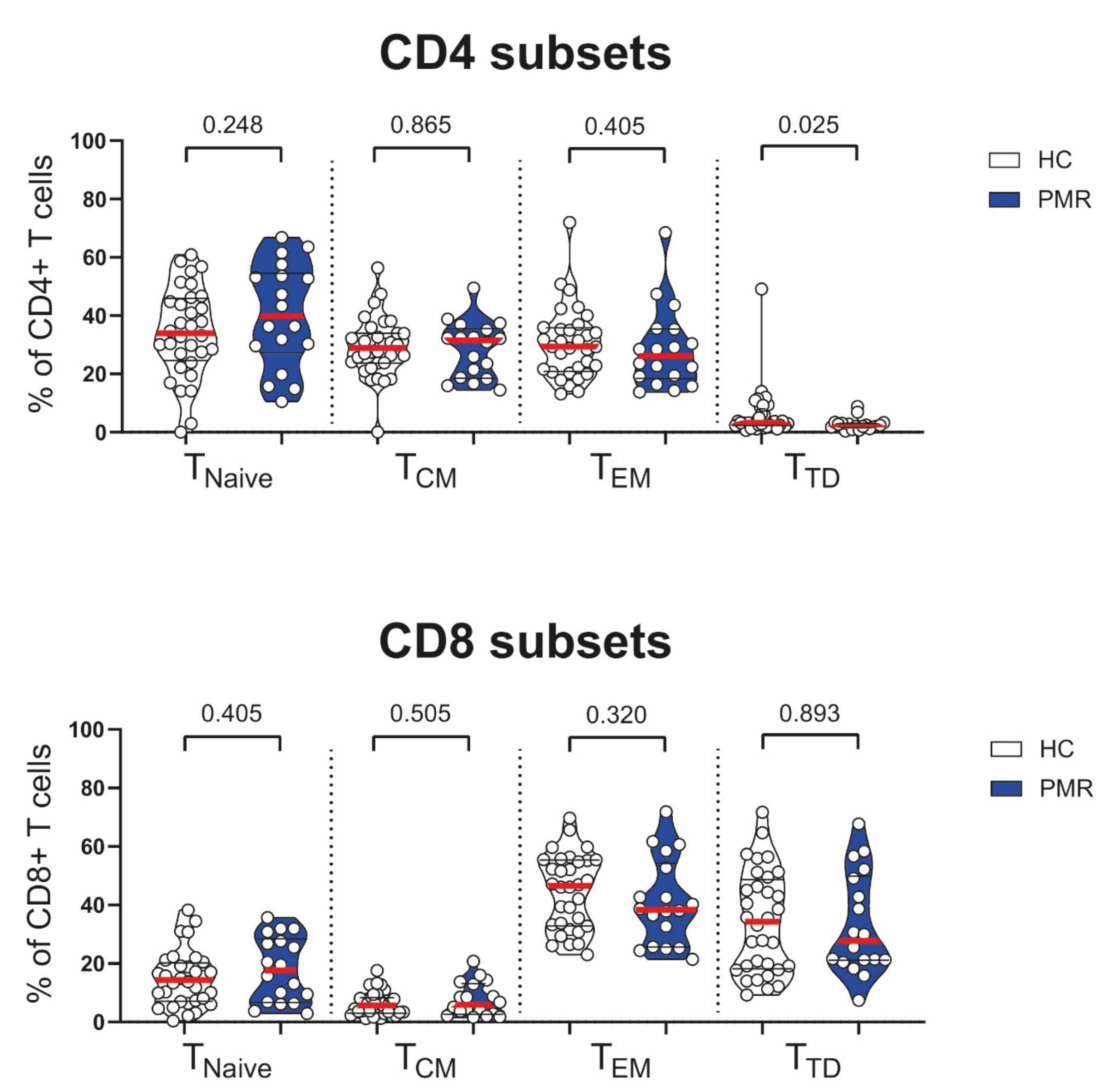

Supplementary Figure 2. IL-4 producing T cells in peripheral blood of patients with PMR and healthy controls. Percentages of CD4^{+}IL-4^{+} (T_{H2}) and CD8^{+}IL-4^{+} (T_{C2}) T cells are shown for 18 patients with PMR and 19 healthy controls. Statistical significance by Mann-Whitney U test is indicated.

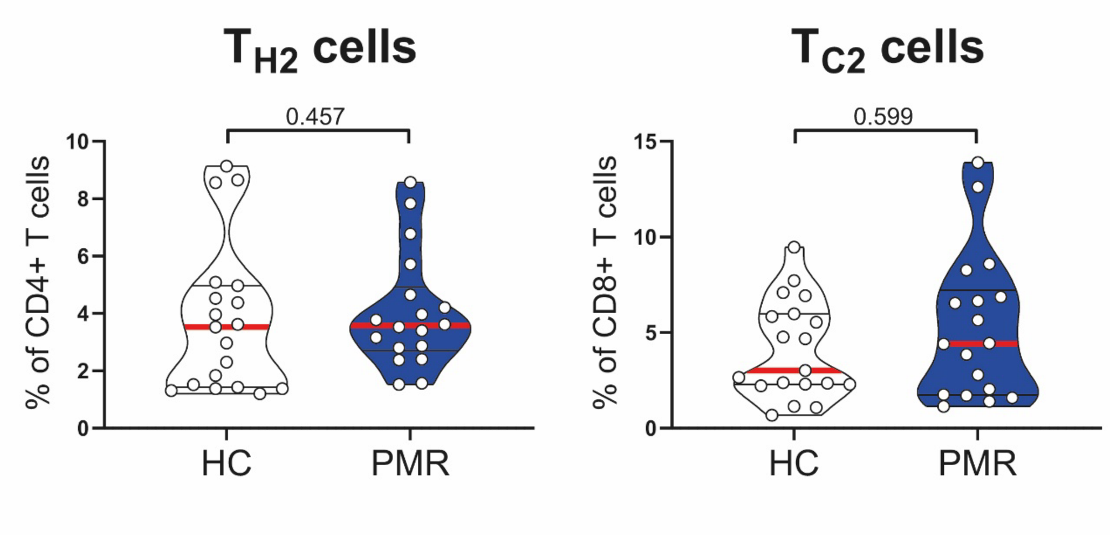

Supplementary Figure 3. Synovial fluid T cell numbers in relation to disease stage. Absolute numbers of $CD3^+$ T cells, $CD4^+$ T cells, $CD8^+$ T cells and $CD19^+$ B cells in peripheral blood (PB) and synovial fluid (SF) of 13 patients with PMR. Symbols indicate whether patients had new-onset or relapsing PMR. Statistical significance by Mann-Whitney U test is indicated. An additional comparison of synovial fluid T cell counts and synovial fluid B cell counts in patients with new-onset PMR versus relapsing disease revealed no differences between these patient groups, as determined by Mann-Whitney U test (data not shown).

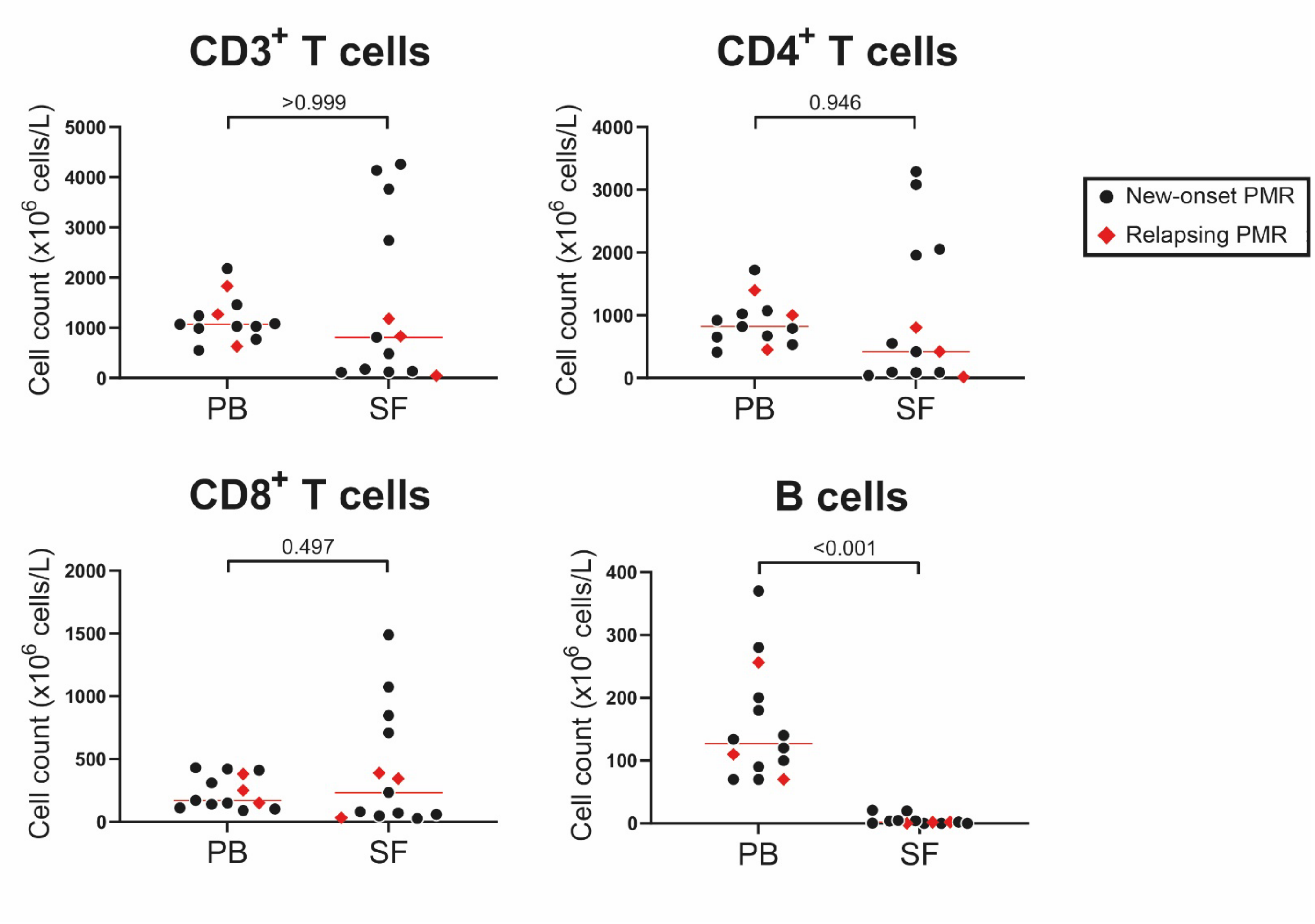

Supplementary Figure 4. T cell differentiation subsets in peripheral blood and synovial fluid of patients with PMR. Percentages of naive (T_{Naive}), central memory (T_{CM}), effector memory (T_{EM}) and terminally differentiated (T_{TD}) CD4^{+} T cells and CD8^{+} T cells in peripheral blood (PB) and synovial fluid (SF) of 9 patients with PMR. Median percentages for CD4^{+} T_{TD} cells were 2.0% (range 0.7 to 3.0) in PB and 0.6 (range 0.1 to 2.5) in SF. Statistical significance by Wilcoxon signed rank test is indicated.

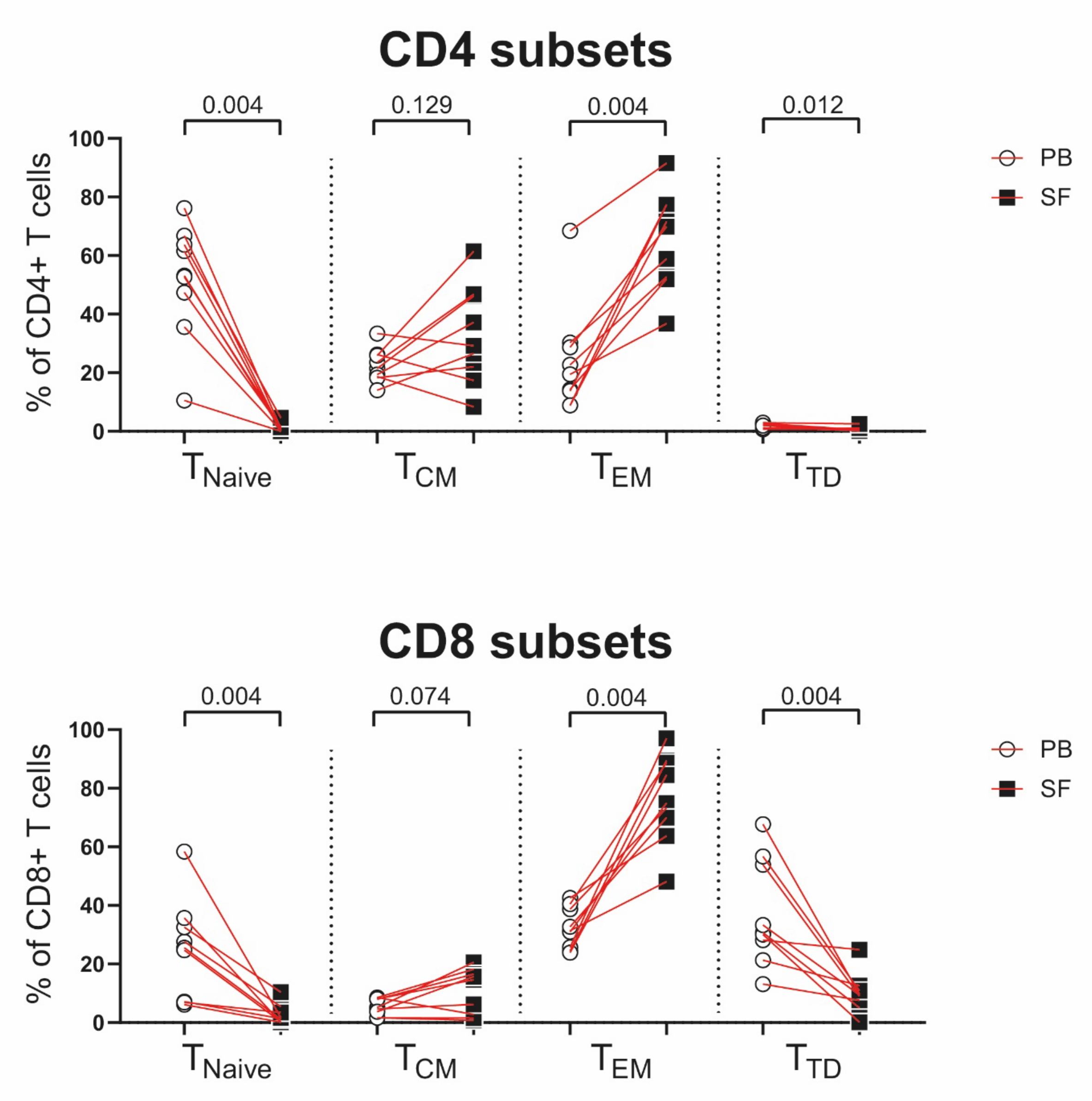

Supplementary Figure 5. **IL-4 producing T cells in peripheral blood and synovial fluid of patients with PMR.** Percentages of CD4$^+$IL-4$^+$ (T_{H2}) and CD8$^+$IL-4$^+$ (T_{C2}) T cells in peripheral blood (PB) and synovial fluid (SF) of 8 patients with PMR. Statistical significance by Wilcoxon signed rank test is indicated.

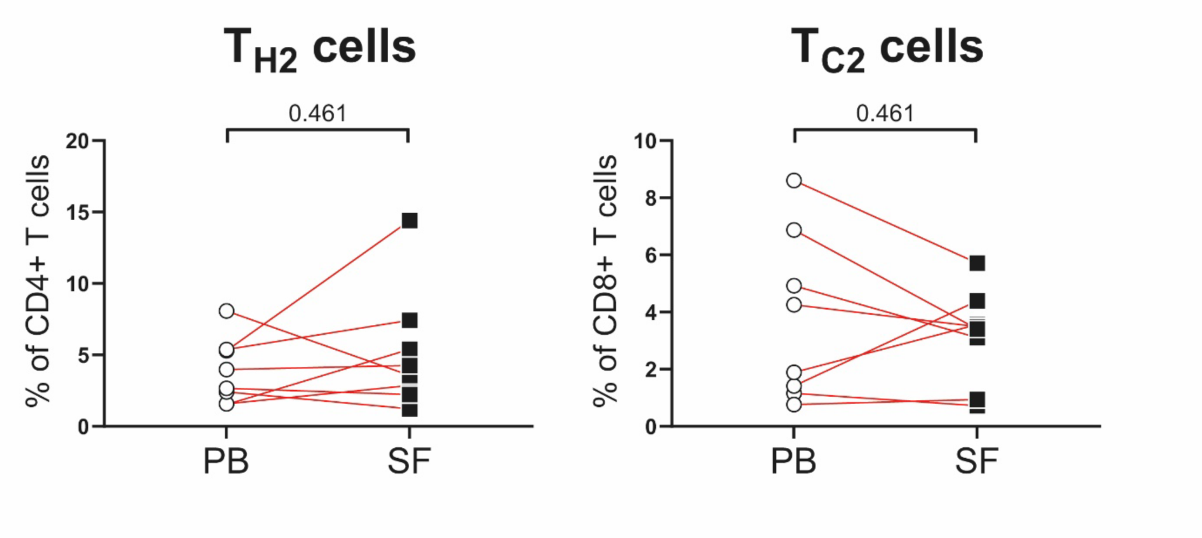

Supplementary Figure 6. IFN-γ and IL-17 production by synovial fluid T cells in relation to disease stage. (A) Percentages of CD4$^+$IFN-γ$^+$IL-17$^-$ (T_{H1}), CD4$^+$IFN-γ$^+$IL-17$^+$ (T_{H1}/T_{H17}) and CD4$^+$IFN-γ$^-$IL-17$^+$ (T_{H17}) T cells, and (B) CD8$^+$IFN-γ$^+$IL-17$^-$ (T_{C1}), CD8$^+$IFN-γ$^+$IL-17$^+$ (T_{C1}/T_{C17}) and CD8$^+$IFN-γ$^-$IL-17$^+$ (T_{C17}) T cells in peripheral blood (PB) and synovial fluid (SF) of 8 patients with PMR. Symbols indicate whether patients had new-onset or relapsing PMR. Statistical significance by Wilcoxon signed rank test is indicated.

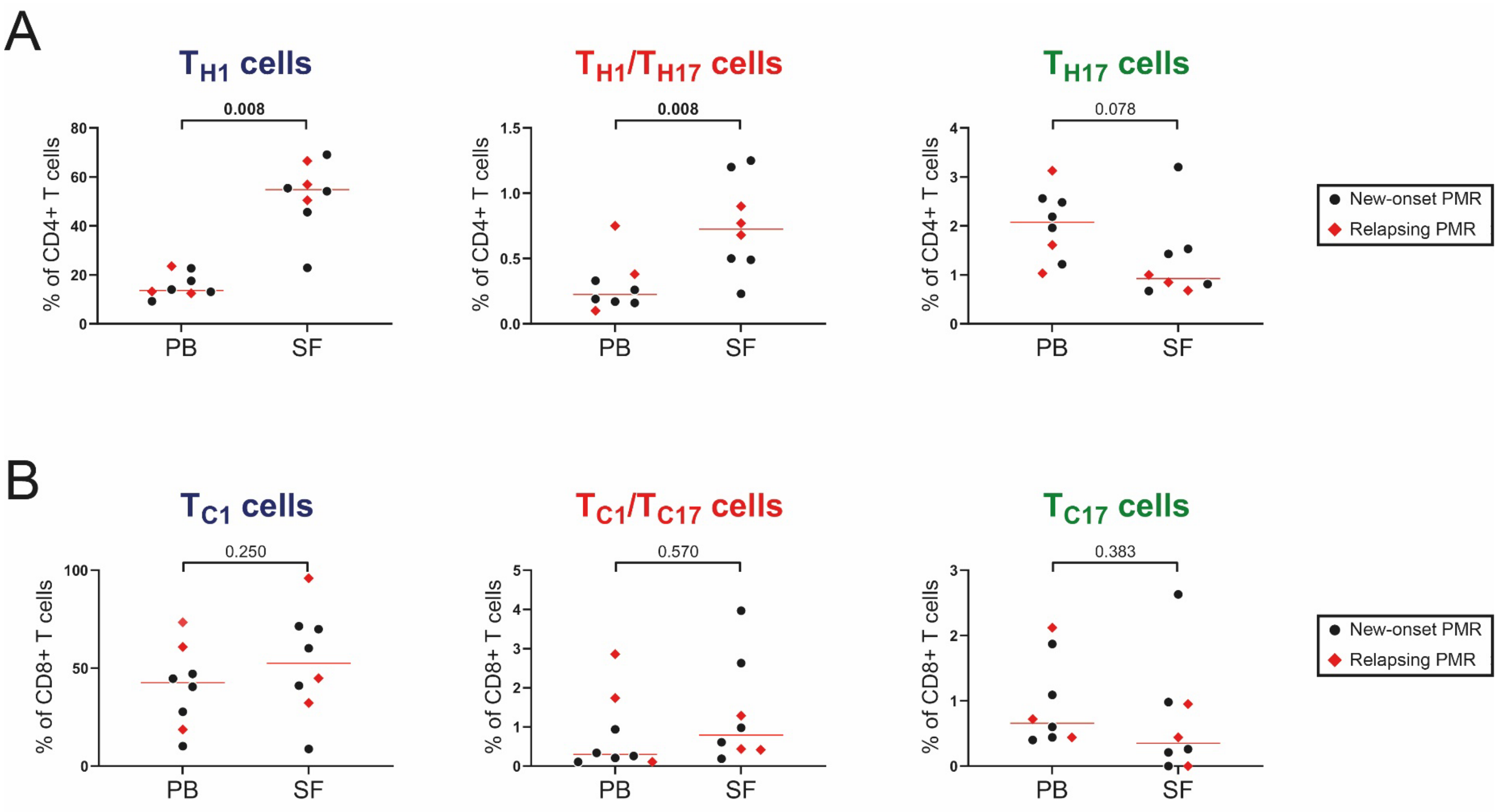

Supplementary Figure 7. Positive tissue controls for immunohistochemistry staining. (A) Immunohistochemistry staining for CD3, CD8, CD20 and CD68 in secondary lymphoid organ tissues and (B) knee synovium obtained from rheumatoid arthritis patients as obtained by mini-arthroscopy.

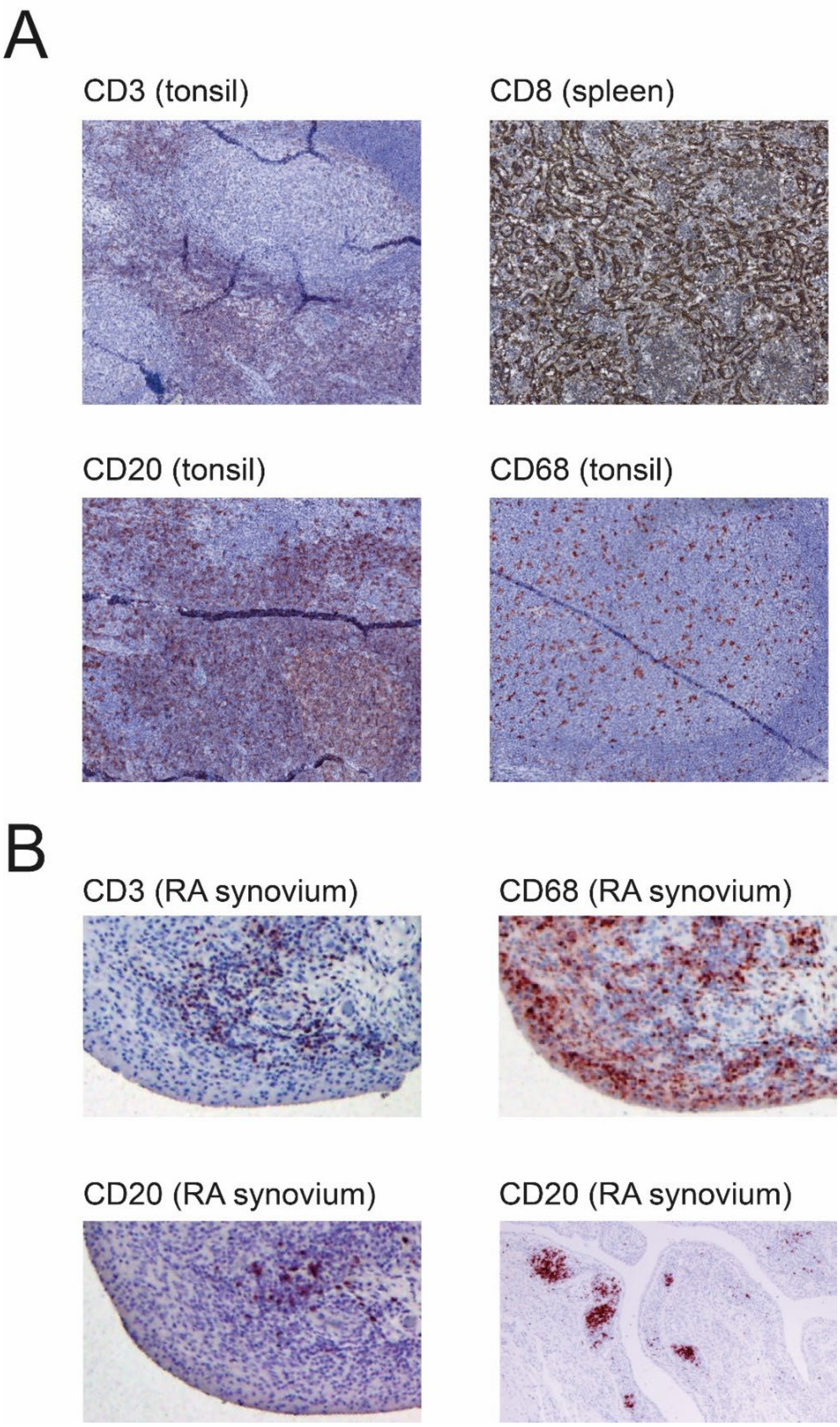

Supplementary Figure 8. Bursa tissue T cells in relation to disease stage. (A) Semi-quantitative scoring for CD3 and CD8 and (B) IFN-γ and IL-17 in SASD bursa tissue biopsies of 11 patients with PMR. Scores of two independent investigators were averaged. Symbols indicate whether patients had new-onset or relapsing PMR.

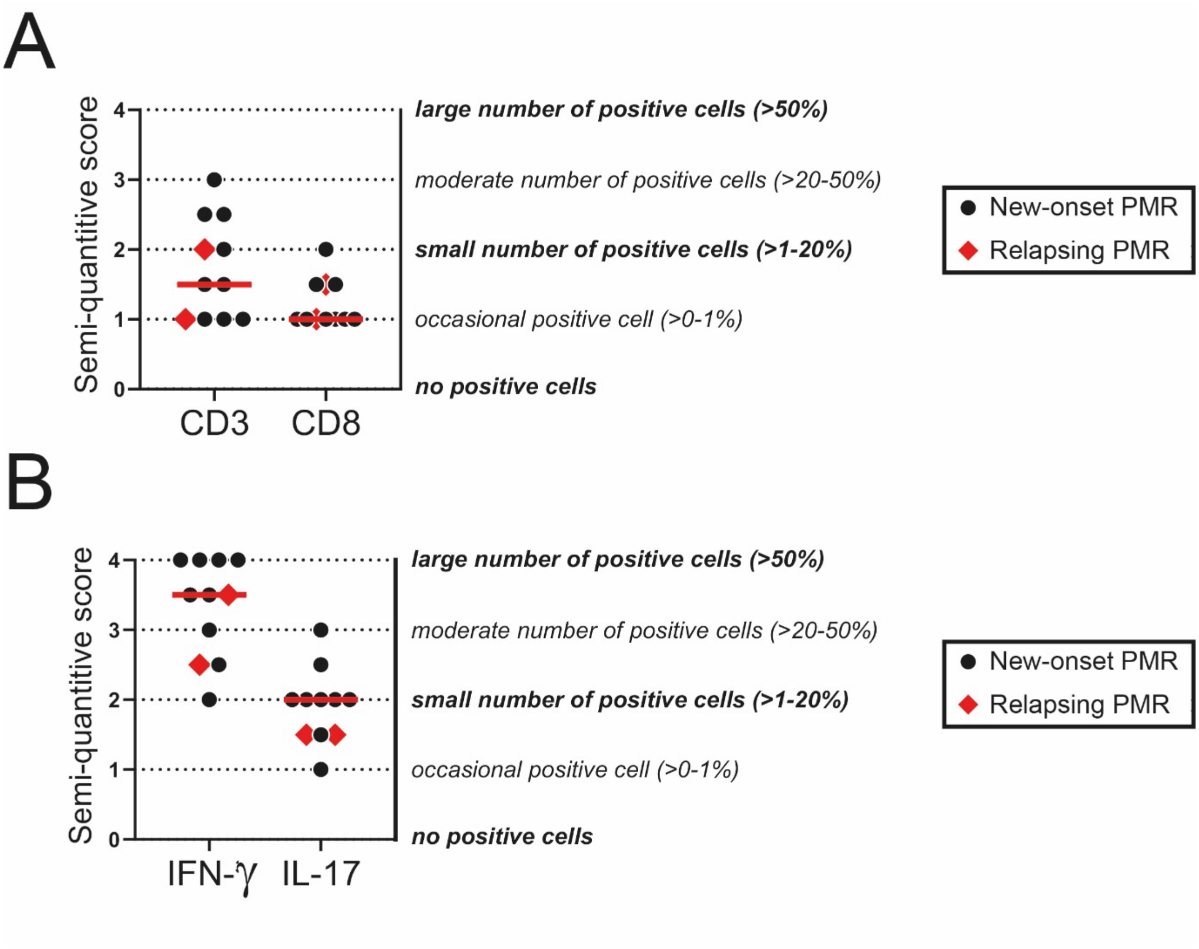

Supplementary Figure 9. Immunofluorescence staining for IFN-γ/CD3 and IL-17/CD3 in synovial biopsies of two additional patients. (A) CD3 (red) and IFN-γ (green) single staining are shown together with DAPI counterstaining (blue). Colocalization of IFN-γ/CD3 is highlighted in yellow. (B) CD3 (red) and IL-17 (green) single staining are shown together with DAPI counterstaining (blue). Colocalization of IL-17/CD3 is highlighted in yellow. Images are shown at 40x magnification.

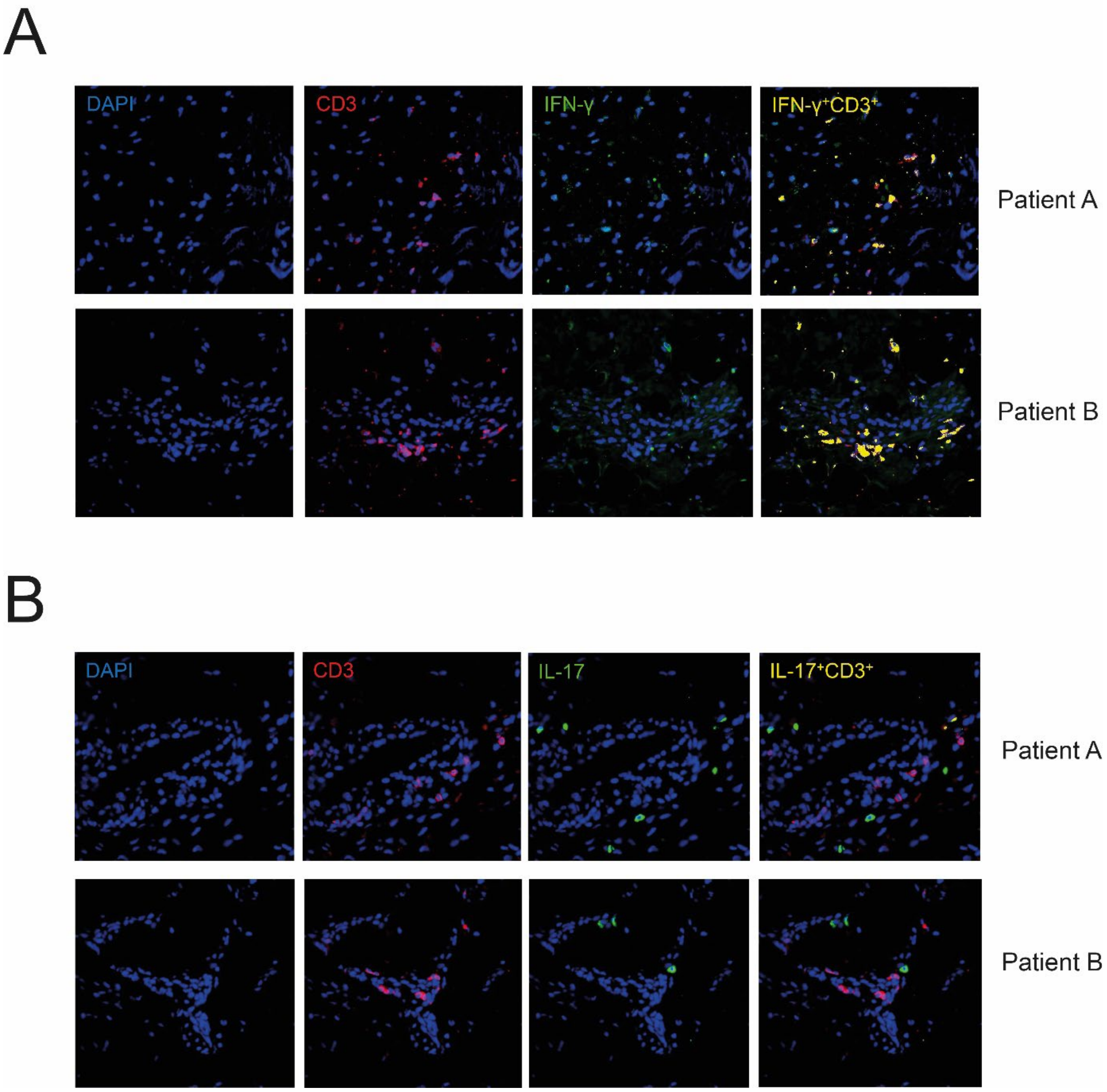

3. References

(1) van der Geest KSM, van Sleen Y, Nienhuis P, Sandovici M, Westerdijk N, Glaudemans AWJM, et al. Comparison and validation of FDG-PET/CT scores for polymyalgia rheumatica. *Rheumatology (Oxford)* (2022) 61:1072-1082. doi: 10.1093/rheumatology/keab483.
